# Olive Bud Dormancy Release Dynamics and Validation of Using Cuttings to Determine Chilling Requirement

**DOI:** 10.3390/plants11243461

**Published:** 2022-12-10

**Authors:** Guillermo Rubio-Valdés, Diego Cabello, Hava F. Rapoport, Luis Rallo

**Affiliations:** 1 Instituto de Agricultura Sostenible, Spanish Council of Scientific Research (CSIC), Alameda del Obispo S/N, 14004 Córdoba, Spain; 2 Excellence Unit ‘María de Maeztu’ 2020-23, Department of Agronomy, Higher Technical School of Agriculture and Forestry (ETSIAM), University of Córdoba, 14071 Córdoba, Spain

**Keywords:** *Olea europaea* L., reproductive budburst, chilling requirements, bearing status

## Abstract

Dormancy release dynamics in olive tree (*Olea europaea* L.) reproductive buds as affected by cold accumulation, tree bearing status, and budburst temperature was studied under natural and controlled conditions, using both cuttings and container- and field-grown plants. The chilling necessary for dormancy release was acquired at different times within the bud population, presenting a progressive pattern of reproductive budburst. Once sufficient chilling is accumulated, 20 °C is a suitable temperature for reproductive budburst, although higher temperature, e.g., 30 °C, during dormancy release can inhibit budburst. While the bearing status of trees determined the amount of return bloom, dormancy release followed a similar pattern for previously bearing and non-bearing trees. Concurrent with investigating budburst factors, the use of shoot cuttings was tested as a method for olive dormancy release studies by contrasting with results from whole trees. It was found it to be valid for studying reproductive budburst, thus providing a useful method to screen chilling requirements in cultivar evaluation and the breeding programs currently ongoing in this species. However, the method was not valid for vegetative budburst, with varying results between cuttings and the whole plant.

## 1. Introduction

A key factor in olive tree, *Olea europaea* L., reproductive development and its well-known biennial bearing cycle is axillary bud dormancy, so understanding the processes of olive bud dormancy onset, maintenance of dormancy, and dormancy release is critical for crop management, selection, and breeding. Furthermore, it is imperative to develop simple and efficient measurement tools to achieve these objectives. This knowledge is especially relevant in current times, with the spread of olive growing to new and climatically diverse areas of cultivation, as well as in considering the global threat of climate change [1].

Bud dormancy of deciduous fruit trees has been extensively studied for many years [2,3,4,5]. Lang [6] proposed the subdivision of plant dormancy into three categories: (1) ecodormancy, when growth is inhibited by external factors; (2) paradormancy, when growth arrest is controlled by physiological factors within the plant external to the dormant organ; and (3) endodormancy, when arrest is under the control of internal physiological factors. However, while it has been generally assumed that the stages of dormancy described in deciduous fruit trees and the responses to the different factors affecting the dormancy period may be similar in evergreens such as the olive tree, there are still uncertainties.

A major difference with deciduous trees, in which reproductive differentiation occurs within the bud prior to the onset of winter dormancy, is that olive tree bud morphology remains undifferentiated as either vegetative or reproductive until dormancy release the following year, when bud reproductive induction and initiation are evident [7,8]. That is, unlike in deciduous fruit trees, there is no morphological evidence of reproductive differentiation in olive axillary buds until budburst, the moment when a previously dormant bud resumes tissue growth [6]. Not until the end of winter, after dormancy release and the initiation of budburst, are vegetative and reproductive axillary bud development clearly distinguished in anatomical observations [8,9,10,11].

Forcing, the placing of plants under beneficial growth conditions, is a useful technique to determine whether winter dormancy has been overcome. This procedure is consistent with the hypothesized entrance of the buds in ecodormancy [6], during which growth is inhibited by external factors such as insufficient warm temperatures. That is, sufficient chilling is assumed to release the buds from endo- and/or paradormancy, but they remain non-growing due to unfavorable environmental conditions, which can be overcome by favorable forcing conditions.

Cuttings provide a valuable experimental system for forcing, as their smaller size in comparison to whole trees facilitates manipulation and repeatability within a relatively small space, especially desirable for trees, where the alternative of using potted trees in growth chambers requires substantial time, space, and financial inversion. In deciduous trees, forcing of cuttings has long been used to test whether winter dormancy has been overcome [5]. In deciduous species, however, the maintenance of cuttings in favorable growth conditions might be simpler, as their budburst comprises the continued growth of already-initiated floral structures, whereas in evergreen trees such as the olive, budburst involves both initiation and continued growth. In the olive tree, cuttings consisting of stem sections with leaves and buds are frequently used for propagation [12,13,14,15], and were first used to evaluate the role chilling in releasing reproductive buds from dormancy by Rallo and Martin [16]. The different organs and tissues which compose the bud, including the subtending leaf [8], may participate to different degrees and at different times in response to flowering controls, and are all contained in the cutting structure.

In agreement with the overall hypothesis that olive tree reproductive budburst requires sufficient winter chilling followed by adequate warm temperature, the experiments presented here intend to characterize the temporal dormancy release pattern of olive tree reproductive buds. Different factors in overcoming this physiological stage, including chilling accumulation, tree bearing status, and the response of budburst to different spring temperatures are considered. Additionally, this study uses both cuttings and potted trees, providing the opportunity to test the use of cuttings as representing the behavior of whole trees.

## 2. Results

### 2.1. Natural and Forced Budburst Following Chilling in the Field

**Figure 1:**
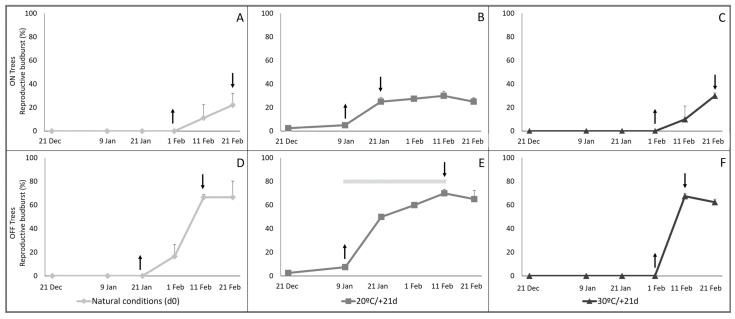
Reproductive budburst following natural chilling in the field of buds present on the trees and on cuttings sampled on the indicated dates and then forced under controlled conditions. Trees were bearing (ON; upper graphs (**A**–**C**)) and non-bearing (OFF; lower graphs (**D**–**F**)). Cuttings were forced in growth chambers at 20 °C and 30 °C, for 21 days (20 °C/+21 d and +30 °C/+21 d). Natural budburst in the field was observed on the sampling date (d0). For OFF, each point represents the average of 240 buds (10 cuttings with 6 buds per 4 trees) in the growth chambers and of 80 buds (20 buds per 4 trees) in the field at d0. For ON, there were 3 trees, so each point represents the average of 180 buds in the growth chambers, 60 buds in the field. Arrows delimit the onset and completion of the budburst period; broad horizontal line in (**E**) shows reference budburst period at 20 °C forcing [8]. Vertical bars = standard error.

As winter chilling increased under natural conditions in the field, progressively greater reproductive budburst occurred for the buds from all treatments, although with different timing and in different amounts. Reproductive budburst started at an earlier field-sample date for the buds on cuttings forced at 20 °C (9 January; Figure 1B,E) than with forcing at 30 °C (1 February; Figure 1C,F). Budburst under natural conditions (Figure 1A,D) was also later than with 20 °C forcing, but final budburst percentage was similar for this and the two forcing temperatures. ANOVA for the budburst period, indicated by the arrows on the Figure 1 graphs, showed a highly significant budburst increase in Figure 1B–F, (*p* = 0.0000), but less significant in A (*p* = 0.1191). For the ON trees, means comparisons of budburst for all dates of each treatment by the Tukey test showed a unique homogeneous group of means for Figure 1A and two significantly different groups in Figure 1B,C. For OFF trees, those comparisons showed two homogeneous groups in Figure 1D, three in Figure 1E, and 2 in Figure 1F. The only mean which overlapped two homogeneous groups corresponded to 2 February in Figure 1E.

Regarding bearing status, total reproductive budburst for the buds from the final sampling date was much higher for OFF (Figure 1D–F) than ON (Figure 1A–C) trees (approximately 65% and 25% of buds, respectively), for all budburst testing conditions (*p* = 0.0000). Moreover, for each forcing temperature, reproductive budburst from ON and OFF trees started on the same sampling date, but under natural conditions appeared to be slightly delayed for ON trees compared to OFF. Still, although budburst percentage was much lower for ON than OFF trees, within each of the three types of observations (natural conditions, 21 days under forcing at 20 °C or 30 °C) the overall temporal patterns were similar for ON and OFF (Figure 1).

**Figure 2:**
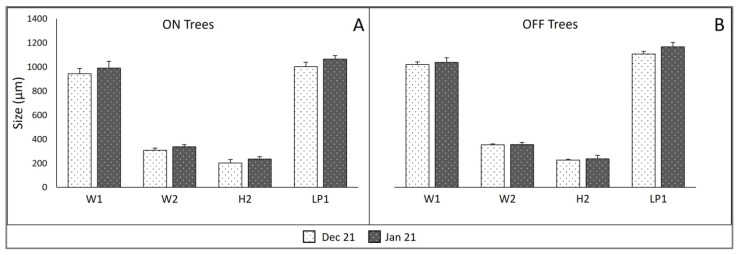
Bud internal dimensions for bearing (ON; (**A**)) and nonbearing (OFF; (**B**)) trees on sample dates 21 December and 21 January. W1 = width of bud base; W2 = width of the bud axis between the primordium axils of the first node; H2 = height between nodes 1 and 3; LP1 = length of the two leaf primordia of the first node. Each column represents the mean of 80 buds (OFF) or 60 buds (ON), based on 20 buds from 10 nodes per tree. Vertical bars = standard error.

Bud dimensions in histological preparations of 21 December and 21 January sampling dates showed similar (no significant differences) bud size for ON trees and OFF trees, and, consequently, no growth occurring between the two dates (Figure 2).

**Figure 3:**
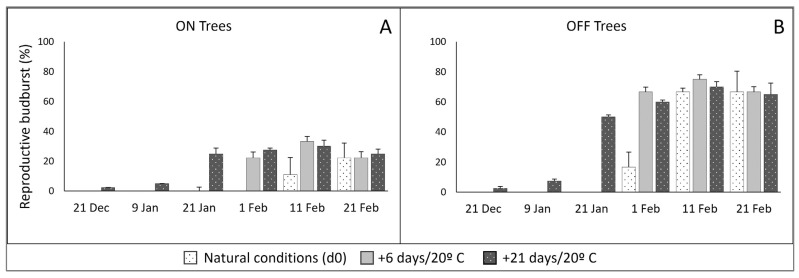
Reproductive budburst percentage from bearing (ON; (**A**)) and nonbearing (OFF; (**B**)) trees sampled at different dates during natural chilling accumulation and observed following three procedures: at the time of sampling (d0), and after growth chamber forcing of cuttings at 20 °C for 6 days (d + 6/20 °C) and 21 days (d + 21/20 °C). Data combine microscope observation of reproductive differentiation and visual observation of phenological stage. Vertical bars = standard error.

In addition, no reproductive differentiation was visible at those times, and the first reproductive differentiation was seen at 11 February (ON) or 1 February (OFF) in the field, evidenced by the d0 observations (Figure 3). In a similar manner, after 6 days at 20 °C forcing, evident reproductive differentiation within the closed buds was first noted in samples from 1 February (Figure 3). It is also interesting to note (Figure 3) that, within each bearing condition, the final reproductive budburst percentages attained showed the same maximum values for all three observation procedures: at the time of sampling (d0), and after forcing of cuttings at 20 °C for 6 days and 21 days.

**Figure 4:**
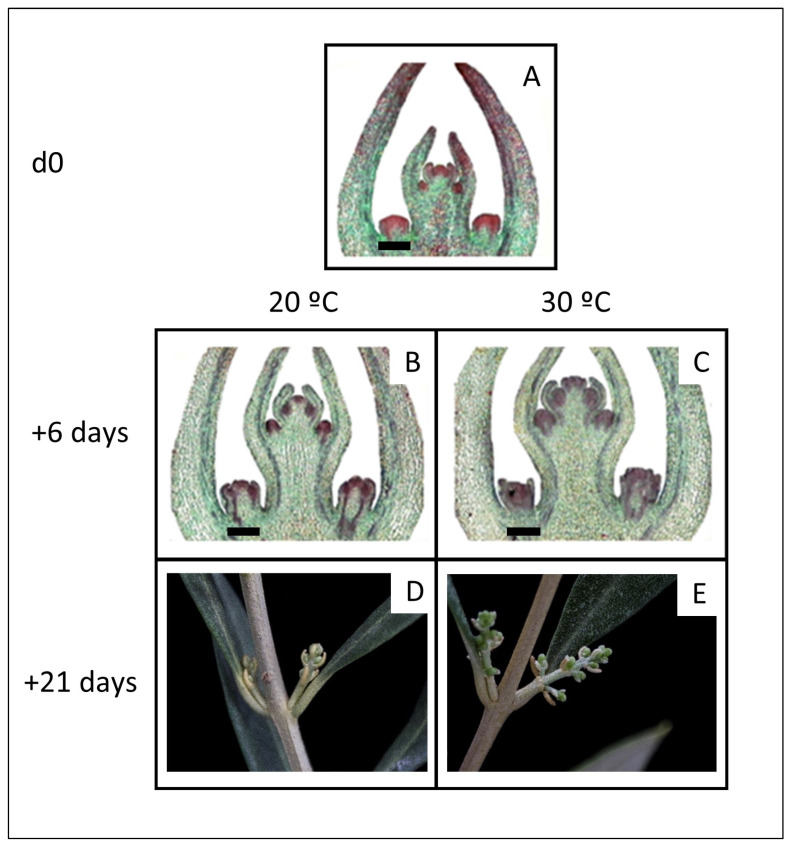
Growth and differentiation of buds sampled 11 February (d0), and after 6 and 21 days of forcing at 20 °C or 30 °C. (**A**–**C**) show central longitudinal histological sections of individual buds, while (**D**,**E**) are photographs of young inflorescences, each developing from one of the two axillary buds at the node. In the histological preparations, reproductive differentiation of the buds is apparent in the darkly staining axillary structures of the buds, particularly within the first, most external pair of bud leaf primordia. Staining described in Section 4.1.2. Bar = 200 µm.

Forcing for 21 days at 30 °C did not yield reproductive budburst until the 11 February field samples (Figure 1C,F). These were much later samples than for 20 °C forcing, in which reproductive budburst was observed in samples from as early as 9 January (Figure 1B,E). Internal histological observation of the buds showed the first definitive reproductive differentiation as increased growth and dense staining in the axils of the first pair of bud primordia, indicating the beginning of inflorescence branch formation (Figure 4A). After six days (+6 d) of forcing at both 20 °C and 30 °C, the buds displayed substantial further development of the axillary structures which will become inflorescence branches and flowers. Furthermore, differentiation of those axillary structures was more advanced at 30 °C than 20 °C (Figure 4B,C). Thereafter, inflorescence development continued more rapidly at 30 °C, and at 21 days of forcing (+21 d) the inflorescences at 30 °C had more nodes and greater extension along their central axes than those forced at 20 °C (Figure 4D,E).

### 2.2. Forced Budburst on Cuttings and Potted Trees Following Cold Accumulation under Artificial Chilling

**Figure 5:**
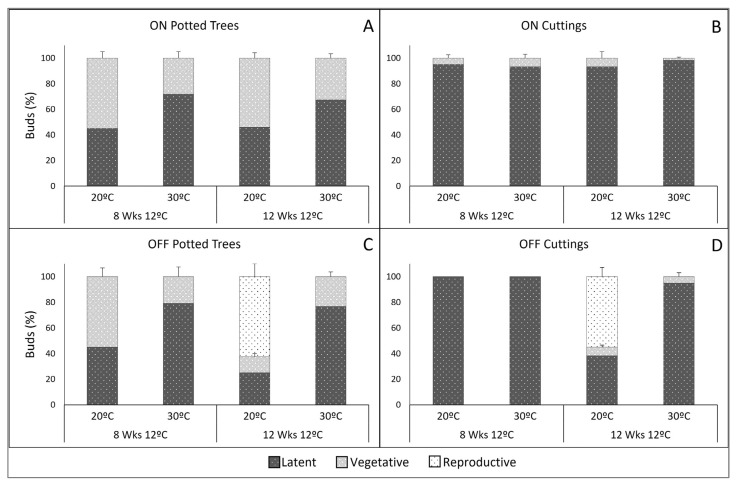
Final bud status (latent, vegetative budburst, or reproductive budburst) after 21 days forcing using (**A**) bearing (ON) potted trees; (**B**) bearing (ON) cuttings; (**C**) nonbearing (OFF) potted trees; and (**D**) nonbearing (OFF) cuttings. All trees received 8 or 12 weeks of cold accumulation in growth chambers at 12 °C. After the cold treatments, forcing at either 20 °C or 30 °C was applied to both cuttings from the trees and to the same whole trees. Each column represents the mean of 240 buds from four trees (60 buds per tree), in the case of both whole trees and cuttings. Vertical bar = standard errors for vegetative and reproductive bud burst percentages.

When potted trees underwent chilling in growth chambers, the results of forcing using cuttings and whole trees were similar for reproductive budburst, but not for vegetative budburst (Figure 5). That is, eight weeks of 10–12 °C cold treatment were insufficient to promote flowering in both of the forcing formats, independent of the tree bearing status. Twelve weeks at 10–12 °C, however, was sufficient to fulfill chilling requirements for reproductive budburst of non-bearing trees for both the potted trees and the cuttings at 20 °C, but not at 30 °C. Furthermore, there was no significant difference between the percentages of inflorescences formed, approximately 60% of the buds, under these two experimental formats (Figure 5C,D).

In contrast to flowering, results for vegetative budburst showed highly significant differences between the two forcing formats. In ON potted trees vegetative shoots formed from approximately 55% of the buds at 20 °C forcing and 25% at 30 °C forcing, after both periods of cold accumulation (Figure 5A). For the OFF trees the percentage of shoot formation was similar to ON for 8 weeks chilling/20 °C forcing, and slightly inferior to ON, for 12 weeks chilling/30 °C forcing (Figure 5C). In the cuttings, this percentage was extremely inferior, reaching at the most 5% (Figure 5D).

## 3. Discussion

### 3.1. Reproductive Budburst Following Chilling Accumulation

Our data illustrate the progression in the percentage of inflorescence emergence as cold accumulated during winter (Figure 1) and the need for the longer of two exposures to artificial chilling in a growth chamber (Figure 5), consistent with previous experimental studies [8,16,17,18,19] and models [20]. The increasing percentages in reproductive budburst with cold accumulation also support the assumption that not all buds on a tree have identical chilling requirements, but each may behave individually [5,21]. Biochemical support for the chilling requirement was presented by Haberman et al. [7] who found that expression of the OeFT1/2 gene in olive leaves and the OeFT2 in buds increased in winter, while initiation of the inflorescences occurred in late winter. Trees artificially exposed to a warm winter expressed low levels of OeFT1/2 in leaves and did not flower. Olive flower induction, therefore, seems to be mediated by an increase in FT levels in response to cold winters.

### 3.2. The Effect of Tree Bearing Condition on Reproductive Budburst

The percentage of flowering buds was much higher for trees which had borne few or no fruit in the fall (OFF) than for trees which had born fruits (ON). Approximately 65% and 25% of buds formed inflorescences for OFF and ON, respectively, in the field study (Figure 1 and Figure 3), and 60% and null for the potted trees (Figure 5). Our data in ‘Arbequina’ are in accordance with the results in ‘Manzanilla de Sevilla’ (Syn. ’Manzanilla’) reported by Rallo and Martin [16] and Ramos [8]. The differences in the amount of flower formation are expected, considering the well-documented alternate bearing behavior of the olive tree, and could relate to competition for assimilates and different inhibitory factors [22,23,24,25]. It is possible that the consistent temporal patterns we observed between ON and OFF trees, in spite of the quantitative differences in budburst percentages (Figure 1 and Figure 3), support the hypothesis of separate controls of flowering level and dormancy release.

### 3.3. High Temperature Inhibition of Reproductive Budburst and Later Stimulation of Differentiation and Growth

When the buds were forced at 20 °C, reproductive budburst started in the 9 Jan samples, while at 30 °C, it was not observed until approximately one month later, in the 11 Feb samples (Figure 1). Nonetheless, many buds had overcome dormancy prior to that time, indicated by the successful budburst at 20 °C forcing (Figure 1B,E; Figure 3). Dennis [26] reported that, in deciduous fruit trees, exposure to high temperature can counteract chilling accumulation and impede dormancy release. Afterwards, the high temperature reversal of the chilling effect may be overcome by further cold accumulation, and the loss of dormancy becomes irreversible [4,27]. This hypothesis, also verified by modeling using chill and heat accumulation overlap [28,29], would explain the previous inactivity at 30 °C forcing, followed by successful reproductive budburst observed for buds sampled after 1 February (Figure 1C,F).

Under the 30 °C forcing conditions, once budburst started (Figure 4A), bud growth and development was much faster. This was seen in both more advanced internal bud differentiation (Figure 4B,C) and faster inflorescence growth (Figure 4D,E) at 30 °C than 20 °C. Thus, overall, while high temperatures inhibit flowering before reproductive differentiation has started, they promote faster growth in the developing inflorescences.

### 3.4. Olive Bud Initial Reproductive Differentiation and Its Relation to Budburst

In this study, no reproductive differentiation of the buds was observed until budburst conditions were fulfilled (Figure 3), consistent with previous anatomical studies showing that dormant buds of the olive tree do not undergo any reproductive differentiation until the release of dormancy and subsequent initiation of budburst [8,9,10]; this has also been verified through scanning electron microscope studies by Haberman et al. [7]. Additionally, the consistent size of the still undifferentiated buds during winter dormancy (Figure 2) is an indication of the lack of growth during that time, confirming previous reports for potentially reproductive buds of cv. Manzanilla [8,10].

The results for progressive sampling dates (Figure 3) and inflorescence differentiation (Figure 4) agree with studies by Ramos et al. [8] using cv. Manzanilla de Sevilla, that once reproductive differentiation starts, it is rapidly followed by inflorescence development. The combining of microscopic observation of internal bud differentiation with the standard visual observation proved useful for more precisely determining the onset of reproductive growth. For general purposes, however, final phenological (visible) observation after 21 days of 20 °C forcing is quite sufficient, as it showed the same reproductive budburst percentage as the earlier time, 6 days, once the maximum was reached (Figure 3).

### 3.5. An Interpretive Scheme for Dormancy Release and Budburst Dynamics in Olive Bud

**Figure 6:**
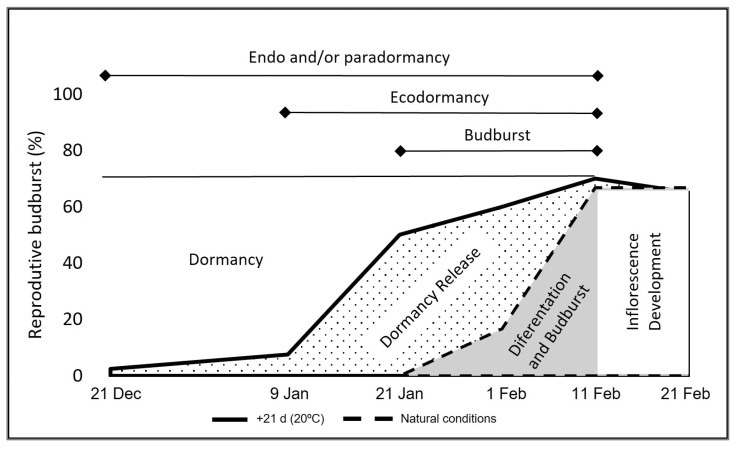
Interpretive scheme for the progression of dormancy phases in the bud population under the study conditions, using OFF tree data. Dates of dormancy release, indicated by reproductive budburst after 21 days forcing at 20 °C, are shown by the solid line. Dates of budburst under natural conditions, for which the buds had to first achieve release from dormancy and then overcome ecodormancy restrictions, are shown by the slashed line. The dormancy periods according to [6] are shown by horizontal lines in the upper part of the figure.

In the scheme presented in Figure 6, the different phases of progressive dormancy release and budburst for the entire bud population are identified, showing the proportion of buds present in each phase at a given time and the phase durations. The case presented uses OFF tree data from Figure 1 to relate the progressive pattern of reproductive budburst after forcing at 20 °C with the progressive pattern of natural budburst (d0). Dormancy release (solid line), is the time when a bud individually has accumulated enough chilling to break dormancy, gaining the capability to initiate reproductive differentiation and growth, thus undergoing budburst when exposed to adequate temperature.

There is a period, between 21 January and 11 February in the conditions of this study, where dormancy release and budburst overlap. That overlap occurs due to the coinciding dormancy-release response to chilling unit accumulation and budburst response to accumulation of heat units, as may occur under natural temperature fluctuation and is demonstrated in modeling these processes [20,28,29].

In the upper part of Figure 6, the horizontal lines indicate periods when the buds are present in the different dormancy phases [6]. All buds were in endo and/or paradormancy at the beginning of the study, and approximately 25% of buds remained in this condition after bud break had fully progressed. Following dormancy release, a progressively greater percentage of buds entered ecodormancy, and starting 21 January, there was a progressive increase in budburst as the ecodormant buds reached adequate warm temperatures in the field to initiate differentiation. Behavior and timing vary among the individual buds, and within the bud population, there can be buds present in different phases at the same time.

### 3.6. Validity of Using Cuttings to Determine Dormancy Release in Reproductive Buds

Shoot-derived cuttings, by assuming dormancy release progresses in the cuttings as in the whole tree, provide a valid organ-level plant structure for testing temperature requirements of dormancy phases of olive reproductive buds. They have been used in dormancy release studies in deciduous fruit trees [2,4,5,30], in forest trees [31], and in olive [8,16,32]. When comparing controlled condition forcing of buds remaining on potted trees and on cuttings taken from those same trees, the percentage of buds undergoing reproductive budburst was the same for both types of experimental configuration (Figure 5). Additional verification for the use of cuttings to study reproductive budburst may be seen in the similar values for final budburst under forcing (cuttings) and natural (on the tree) conditions (Figure 1 and Figure 3).

To best standardize the cutting methodology for studying olive reproductive budburst, our results highly recommend obtaining the cuttings from trees in OFF (non-bearing). Even though the temporal pattern for dormancy release was similar for ON and OFF trees, reproductive budburst levels were much lower or even absent in ON trees (Figure 1, Figure 3 and Figure 5), while the proportionally higher levels in OFF trees provide more reliable information. It is also possible to “produce” nonbearing trees by removing the reproductive structures.

In contrast to reproductive budburst, the cutting system was not found to be consistent for determining vegetative budburst, as very large differences were observed compared to the buds on whole potted trees (Figure 5). Vegetative budburst in the cuttings was extremely low or absent, whereas on the trees, it occurred in up to 65% of buds and showed consistent tendencies for similar treatments (Figure 5). It is unclear why those differences occurred, but one possible hypothesis is that, while reproductive buds accumulate chilling units to break dormancy within the bud itself or the subtending leaf [7,8], in vegetative buds the control might come from a source outside the cutting.

## 4. Materials and Methods

This study comprises two different experiments that consisted of an initial cold treatment of variable duration that was supplied either naturally (outdoors on fully-grown mature trees, experiment 1) or artificially (on potted trees in a growth chamber, experiment 2). Following different periods of cold treatment, the buds, present either on cuttings (both experiments) or on the potted trees (experiment 2), were forced in climatic chambers at 20° (standard temperature for bud growth once dormancy has been overcome) or 30 °C (a representative high Spring temperature). Temperatures and 21-day total forcing period were indicated by results of Ramos et al. [8]. The experiments were conducted with ‘Arbequina’, currently one of the most grown cultivars worldwide, and, furthermore, useful for experiment 2 because it has a short juvenile period, thus initiating reproductive behavior at an early age and small size.

### 4.1. Experiment 1: Bud Behavior Following Cold Accumulation of Trees under Field Conditions

#### 4.1.1. Organization of Experiment 1

In fall–winter season, six field-grown adult (seven-years-old) olive trees cv. Arbequina were chosen at the IFAPA (Andalusian Institute of Agricultural and Fisheries Research and Training) at the Alameda del Obispo Center in Córdoba, Spain, three in ON and four in OFF bearing status. Temperatures registered in the IFAPA weather station during the experiment were typical for the experimental site, ranging from 0.5 °C minimum to 20.8 °C maximum in January, and from 13.1 °C to 20.3 °C in February.

**Figure 7:**
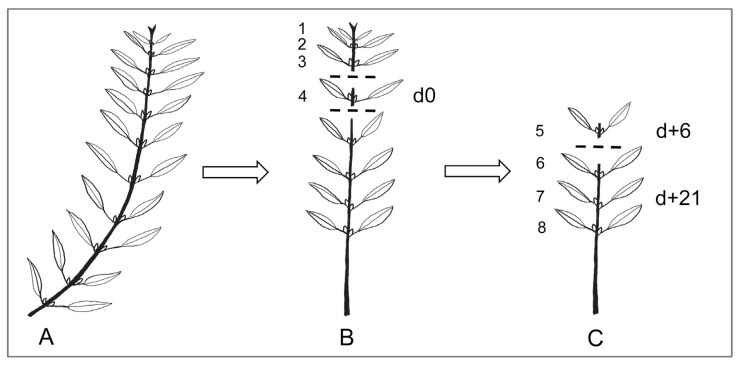
Procedure to obtain cuttings and samples for anatomical observations. (**A**) Shoots with at least 8 nodes are sampled from the tree. (**B**) The 3 distal (tip) nodes and the leaves and buds below node 8 are removed. Node 4 is fixed on the initial date (d0) for later microscopic observation, and cuttings containing nodes 5–8 are forced under controlled conditions. (**C**) After 6 days (d + 6) forcing, node 5 is fixed. The cuttings, now containing 3 nodes, are maintained under forcing until a total of 21 days (d + 21).

Testing budburst by forcing cuttings was carried out as follows. The olive-tree buds are located in the axils of the two opposite leaves at each node of current-year shoots (Figure 7). At successive times during winter, shoots with at least eight nodes (Figure 7A) were excised from around the central canopy. On the day of sampling (d0), the first three apical nodes and all leaves below node 8 were removed, and node 4 was excised and preserved in FAE (formalin: acetic acid: 60% ethanol (2:1:17 *v*/*v*/*v*); Jensen [33]) for microscope observation of bud development (Figure 7B). The shoot cuttings, now consisting of four leafy nodes above the denuded portion of the stem, were then forced in controlled-climate chambers. After 6 days (d + 6) forcing, the most apical node of each cutting (node 5 of the original shoot) was preserved in FAE (Figure 7C) as above. The cuttings, now with three remaining leafy nodes (nodes 6, 7, and 8 of the original shoot) were left in the chamber until d + 21 (Figure 7C), when final visual observations [34] were made of all six axillary buds per cutting. In summary, microscope observations were made of one-node (two-bud) samples on the day of field sampling (d0) and following 6 days of forcing, and visual observations made after 21 days of forcing.

The cuttings were sprayed with copper sulfate (CuSO_4_) at 200 ppm for fungal control and placed in plastic trays containing perlite in the climatic chambers at 20 °C and 30 °C, with 12/12 h day/night photoperiod, and photon flux density of 200 μmol m^−2^ s^−1^. Ten cuttings per tree were evaluated for each of the six (21 December, 9 January, 21 January, 1 February, 11 February and 21 February) sampling dates and two spring forcing temperatures (20 °C and 30 °C). In addition, fifteen similar shoots were tagged on each tree as controls at the beginning of the experiment to observe budburst under natural conditions.

We used a broadened definition for reproductive budburst, in which we combined microscopic observation of internal bud differentiation prior to externally visible growth with the standard use of visible phenological stages [34]. In this way, in addition to obtaining a more precise indication of the onset of reproductive growth, we integrated the observations of both closed and visibly growing buds.

#### 4.1.2. Histological Preparation and Observations

Closed buds of d0 and d + 6 were processed for histological observations. Following dehydration and embedding in paraffin the buds were sectioned longitudinally at 12 μm and stained with Tannic acid–Ferric chloride–Safranin-Fast green [33]. Orienting the sample in relation to the decussate phyllotaxy of the olive tree, we sectioned in the plane of bud nodes 1, 3, and 5, and used the central longitudinal sections to observe bud internal structure and to evaluate whether a bud had initiated reproductive differentiation [8,10].

To detect any bud growth during winter dormancy, the d0 (field sampling day) buds for 21 December and 21 January were measured in the central longitudinal sections, using an optical microscope equipped with image analysis (VIDS V; Ai Cambridge, Pampisford, U.K.). Eighty buds (20 buds per tree) for the four trees of each bearing status were measured as by [10]. Width of the bud base (W1), width of the bud axis at first node (W2), and axis length between nodes 1 and 3 (H2) were determined. In addition, the internal lengths of the two leaf primordia of the first (most external) node (LP1) were included. The data were processed by ANOVA and mean comparison for each sampling date was performed (more details in Section 4.3).

### 4.2. Experiment 2: Bud Behavior in Potted Trees Following Cold Accumulation under Artificial Chilling and Comparison with the Use of Cuttings

The experiment employed 32 three-year-old olive trees cv. Arbequina planted in 14 L pots with abundant flowering were selected. In spring, all inflorescences were removed from half of them for their use as non-bearing trees, while the inflorescences of the other 16 trees were left to set fruit and the trees used as bearing trees. On 1 September, all trees were placed in a climatic chamber at constant 10–12 °C temperature of for cold accumulation and the trees were split in two groups for two cold-treatment durations (8 weeks or 12 weeks). At the end of the cold treatments, ten shoots at least eight nodes long were selected from the central canopy zone of each tree. Five of these shoots were used to prepare 3-node cuttings as described for experiment 1 (Figure 7), and the cuttings were placed in plastic trays with vermiculite as before. The other five shoots were tagged, maintained on the tree, and their apical three nodes were removed to increase similarity to the cuttings. Both the potted trees and the cuttings in trays were put in climatic chambers at either 20 °C or 30 °C for forcing during 21 days, at which time reproductive and vegetative budburst [34] were recorded. The behavior of shoots maintained on the tree was compared to that of the cuttings of the same trees to validate or discard the use of cuttings for the study of reproductive and vegetative budburst.

### 4.3. Statistical Analysis

Experiments 1 and 2 evaluated the reproductive budburst response to chilling accumulation in trees with either ON or OFF bearing status. In experiment 1, there were three (ON trees) and four (OFF trees) replications for each of the six sampling dates. In experiment 2, there were four trees and four groups of five cuttings for each of the eight forcing groups: two bearing levels, (ON or OFF), two periods of treatments (8 or 12 weeks), and two forcing temperatures (20 and 30 °C).

For statistical analysis, we used the Statistix Program for Windows (Version 9.0). To determine the progression of reproductive budburst, we tested the data for normality using the Shapiro–Wilk test and then ANOVA carried out as data distribution was normal. In experiment 1, when differences were significant for sampling dates, separation of means between budburst dates was performed using the Tukey test, while in experiment 2, comparison of means was used.

## 5. Conclusions

The results characterize the temporal pattern of dormancy release of olive tree reproductive buds, following chilling accumulation, and expand knowledge regarding factors involved. Using both natural (field) conditions and forcing at different temperatures in controlled chambers, the percentage of flowering buds was much higher for nonbearing than bearing trees but the pattern of reproductive budbreak over time was consistent. Once sufficient chilling has been accumulated, 20 °C forcing was confirmed suitable for normal reproductive budburst; when buds had just barely overcome dormancy, however, high (30 °C) temperature inhibited budburst, but that did not occur on later dates, after buds had initiated reproductive differentiation. We present an illustrative scheme to summarize the pattern of release from dormancy and reproductive budburst in olive buds.

It is important to determine chilling requirements for dormancy release and subsequent flowering for the selection and breeding of appropriate olive genotypes, particularly to identify those with low requirements, and, thus, be able to maintain productivity under climate warming and in new, mild-winter growing areas. By comparison with forcing whole trees and with budburst in the field, forcing of cuttings was verified as suitable for testing olive reproductive but not vegetative budburst. The use of cuttings, representing valuable savings in time, space and financial inversion, can now be solidly recommended for olive tree cultivar selection and breeding. Furthermore, the results presented here not only provide specific information regarding experimental procedures, but also indicate relevant parameters, such as dates of onset and completion of budburst, and the length of the budburst period.

## Data Availability

Not applicable.
